# Loss of interferon regulatory factor 5 (IRF5) expression in human ductal carcinoma correlates with disease stage and contributes to metastasis

**DOI:** 10.1186/bcr3053

**Published:** 2011-11-04

**Authors:** Xiaohui Bi, Meera Hameed, Neena Mirani, Erica Maria Pimenta, Jason Anari, Betsy J Barnes

**Affiliations:** 1 Department of Biochemistry & Molecular Biology, New Jersey Medical School, UMDNJ, 185 S. Orange Ave., Newark, NJ 07101, USA; 2 New Jersey Medical School-University Hospital Cancer Center, UMDNJ, 205 S. Orange Ave., Newark, NJ 07103, USA; 3 Department of Pathology, New Jersey Medical School, UMDNJ, 185 S. Orange Ave., Newark, NJ 07101, USA; 4 Department of Pathology, Memorial Sloan-Kettering Cancer Center, 1275 York Ave., New York, NY 10065, USA; 5 UMDNJ-Robert Wood Johnson Medical School, 675 Hoes Lane, Piscataway, NJ 08854, USA

## Abstract

**Introduction:**

New signaling pathways of the interleukin (IL) family, interferons (IFN) and interferon regulatory factors (IRF) have recently been found within tumor microenvironments and in metastatic sites. Some of these cytokines stimulate while others inhibit breast cancer proliferation and/or invasion. IRFs, a family of nine mammalian transcription factors, have multiple biologic functions that when dysregulated may contribute to tumorigenesis; most well-known are their roles in regulating/initiating host immunity. Some IRF family members have been implicated in tumorigenesis yet little is still known of their expression in primary human tumors or their role(s) in disease development/progression. IRF5 is one of the newer family members to be studied and has been shown to be a critical mediator of host immunity and the cellular response to DNA damage. Here, we examined the expression of IRF5 in primary breast tissue and determined how loss of expression may contribute to breast cancer development and/or progression.

**Methods:**

Formalin-fixed paraffin-embedded archival breast tissue specimens from patients with atypical ductal hyperplasia (ADH), ductal carcinoma *in situ *(DCIS) and invasive ductal carcinoma (IDC) were examined for their expression of IRF1 and IRF5. Knockdown or overexpression of IRF5 in MCF-10A, MCF-7 and MDA-MB-231 mammary epithelial cell lines was used to examine the role of IRF5 in growth inhibition, invasion and tumorigenesis.

**Results:**

Analysis of IRF expression in human breast tissues revealed the unique down-regulation of IRF5 in patients with different grades of DCIS and IDC as compared to IRF1; loss of IRF5 preceded that of IRF1 and correlated with increased invasiveness. Overexpression of IRF5 in breast cancer cells inhibited *in vitro *and *in vivo *cell growth and sensitized them to DNA damage. Complementary experiments with *IRF5 *siRNAs made normal mammary epithelial cells resistant to DNA damage. By 3-D culture, IRF5 overexpression reverted MDA-MB-231 to normal acini-like structures; cells overexpressing IRF5 had decreased CXCR4 expression and were insensitive to SDF-1/CXCL12-induced migration. These findings were confirmed by *CXCR4 *promoter reporter assays.

**Conclusions:**

IRF5 is an important tumor suppressor that regulates multiple cellular processes involved in the conversion of normal mammary epithelial cells to tumor epithelial cells with metastatic potential.

## Introduction

Breast cancer is a heterogenous disease whose progression from atypical ductal hyperplasia (ADH) to ductal carcinoma *in situ *(DCIS) and invasive ductal carcinoma (IDC) is regulated by the aberrant expression of multiple mediators produced by the mammary tumor itself and the adjacent reactive stroma [1]. These signals promote tumor cell proliferation, survival, establishment of tumor vasculature, invasion and ultimately metastasis to secondary organs. The ability of the tumor to create a state of local immune suppression allows tumor cells to evade clearance by the immune system [2]. Signaling pathways that regulate cytokine/chemokine expression (ILs, IFNs and interferon regulatory factors (IRFs)) have recently been found within tumor microenvironments and in metastatic sites; some of these cytokines stimulate while others inhibit breast cancer proliferation and/or invasion [2]. The role of these cytokines in disease progression, as markers of disease stage, and as novel treatment strategies requires further attention.

IRF5 is a transcription factor that regulates type I IFN signaling [3] and cytokines/chemokines with lymphocyte-chemotactic activities, that is, RANTES, MIP1α/β, MCP1, I309, IL8 and IP10 [4]. Subsequent studies demonstrated its critical role(s) in the cellular response to extracellular stressors including virus, DNA damage, Toll-like receptor (TLR) and death receptor signaling [3-11]. Depending on the cell type, loss of *IRF5 *yields cells incapable of a sufficient immune response to pathogens and/or undergoing apoptosis [6,8-11]. Northern blot analysis of *IRF5 *tissue-specific expression revealed that it is primarily expressed in lymphoid tissues but can be induced in multiple cell types [3,12,13]. IRF5 has been associated with the regulation of important cellular processes, such as cell growth, apoptosis, cell cycle arrest, and cytokine production [6-9,14].

Little is known of IRF5 tumor suppressor function. *IRF5 *was mapped to chromosome 7q32 [3] that contains a cluster of imprinted genes and/or known chromosomal aberrations and deletions in lymphoid, prostate, and breast cancer [15-22]. *IRF5 *expression is absent or significantly decreased in immortalized tumor cell lines and primary samples from patients with hematological malignancies, suggesting for the first time its role as a tumor suppressor gene [3,7]. Recent data from *irf5^-/- ^*mice support its candidacy as a tumor suppressor gene [9]. Mouse embryonic fibroblasts (MEFs) from *irf5^-/- ^*mice are resistant to DNA damage-induced apoptosis and can be transformed by *c-Ha-ras *[9]. Conversely, ectopic expression suppresses malignancy of cancer cell lines *in vitro *and *in vivo *[7,23]. While IRF5 has been shown to be a direct target of p53 [23], data from our lab and others indicate that IRF5 acts on an apoptotic signaling pathway that is distinct from p53 [7-9].

Loss of tumor suppressor genes represents a critical event in the development and progression of breast cancer. However, while an increasing number of oncogenes have been identified in breast cancer, few tumor suppressor genes have been directly implicated in the development/progression of this disease. Altered expression or function of tumor suppressor genes *BRCA1*, *BRCA2 *and *p53 *do not fully account for the high prevalence of spontaneous breast cancers. Loss or mutation of *BRCA1 *occurs in < 10% of all breast cancers, while *p53 *is mutated in up to 30% of breast cancers [24]. There are likely other tumor suppressor genes and oncogenes contributing to breast tumorigenesis. IRF1 was recently shown to have tumor suppressor function in breast cancer, while increased expression of IRF2 was associated with oncogenic activation [25]. Overexpression of IRF1 induced apoptosis and inhibited tumor growth in mouse and human mammary cancer cells [26-28]. The focus of the present study was to examine and compare IRF1 and IRF5 expression in human breast tissue and to determine whether IRF5 acts as a tumor suppressor. Data presented here support a unique role for IRF5 in regulating mammary epithelial cell growth and provide the first direct evidence that loss of IRF5 tumor suppressor function contributes to breast tumorigenesis.

## Materials and methods

### Cell lines and culture

Human immortalized breast cells MCF-12A, MCF-7, MDA-MB-231, -436, -468, and T47D were purchased from American Type Culture Collection (Manassas, VA, USA) in spring 2009, and aliquots were frozen in liquid nitrogen until time of use. Cells were cytogenetically tested and authenticated (by STR profiling from ATCC) before freezing. The amphotrophic helper-free Phoenix cells were provided by G. Nolan (Stanford, CA, USA). All breast cancer cells lines and 293T-derived Phoenix cells were propagated in Dulbecco's modified Eagle's medium (Sigma-Aldrich, St. Louis, MO, USA) containing 10% fetal bovine serum (Sigma) and 1 IU penicillin/1 μl/ml streptomycin (Mediatech, Hemdon, VA, USA) at 37°C in a humidified incubator with 5% CO_2_/95% air. MCF-12A were grown in DMEM F-12 supplemented with 5% horse serum (Sigma), 100 ng/ml cholera toxin (Sigma), 20 ng/ml EGF (Invitrogen, Carlsbad, CA, USA), 10 μg/ml insulin (Sigma), and 500 mg/ml hydrocortisone (Sigma). Each vial of frozen ATCC authenticated cells was thawed and maintained in culture for a maximum of six weeks. There were enough frozen vials for each cell line to ensure that all experiments were performed on cells that had been tested and in culture for six or more weeks.

### Chemicals and treatments

Doxorubicin was from Sigma; Interferon (IFN)-γ from PBL InterferonSource (Piscataway, NJ, USA). Cells were treated with 0.1 or 1 μM Doxorubicin or 1,000 U/ml IFN-γ for the indicated time periods. Cells were exposed to 2, 5 or 10 Gray (Gy) of ionizing radiation (IR) using a self-shielded Cs-137 irradiator.

### Retroviral construction and transduction

IRF5 was cloned into the pBabe-puromycin vector at BamHI/SalI sites transfected to Phoenix cells as described [29]. Viral supernatants were collected 48 h post-transfection and used to infect MCF-7, MDA-MB-231 and -468 cells. After two days, media was exchanged for puromycin selection to obtain stable transfectants. Cultures were pooled from each cell line and positive infection determined by Western blot with mouse anti-IRF5 antibodies (M01, Novus Biologicals, Littleton, CO, USA).

### Immunofluorescence (IF), immunohistochemistry (IHC) and semi-quantitative evaluation

H&E sections of formalin-fixed paraffin-embedded (FFPE) archival tissue specimens were reviewed by two pathologists (MH and NM) for histological evaluation of disease and grade. Slides from 19 patients with ADH, 24 with DCIS, 29 with IDC, and 11 with lymph node metastases were evaluated for IRF expression. Normal breast tissue from the same donors or adjacent to tumors were characterized in 51 patients. Sections were obtained from the Pathology Department at UMDNJ New Jersey Medical School (NJMS). The study was approved by the NJMS Institutional Review Board (IRB) and all participants provided written informed consent. Antigen retrieval was performed by heating slides at 95°C in citrate buffer (pH 6.0) for one hour before staining with mouse anti-IRF5 or rabbit anti-IRF1 (C-20, Santa Cruz Biotechnology, Santa Cruz, CA, USA) antibodies at 1:100 dilution in 4% BSA overnight. For IF, slides were incubated with anti-rabbit Cy3 and anti-mouse FITC (Molecular Probe, Eugene, OR, USA) antibodies at 1:1,000 in 4% BSA/PBST. Slides were mounted with DAPI mounting buffer (Vector Laboratories, Burlingame, CA, USA) and images captured on a Zeiss Axiovert 200 fluorescent microscope; quantification was performed using Axiovision software (Carl Zeiss Microimaging, Oberkochen, Germany). For IHC, slides were incubated with 1:200 diluted anti-IRF5 for two hours then 1:1,000 diluted Alkaline Phosphatase anti-mouse IgG (Vector Laboratories, AP-2000) and developed with the Vector^® ^Blue Alkaline Phosphatase (BAP) Substrate Kit III (Cat. No. SK-5300). The second staining was with 1:200 diluted anti-IRF1, Peroxidase anti-Rabbit IgG (Vector Laboratories, PI-1000) and developed with DAB Substrate Kit (Vector Laboratories, SK-4100). The nucleus was stained with Nuclear Fast Red mounting buffer.

Evaluation of stained slides was assessed by one pathologist (MH) and two independent reviewers (XB and BJB or JA), who were unaware of the patient's characteristics. Two slides from different areas of the same tumor were examined and scored independently by each reviewer with a consensus being reached in difficult cases (< 5% for each antibody). Following initial review, an arbitrary grading system was defined for each antibody in which the density of positive cells within normal ducts and lobules or ADH, DCIS and IDC as defined by the tumor (and not the stroma) was assessed semi-quantitatively on the whole tissue section. This classification allowed the stratification of the tumors as positive or negative for IRF1 and IRF5.

### Western blotting

Preparation of cellular lysates and immunoblotting were performed as described [30,31]. Proteins were transferred to nitrocellulose membrane and detected with horseradish peroxidase (HRP)-conjugated secondary antibody (1:2,000) followed by enhanced chemiluminescence (Amersham Biosciences, Piscataway, NJ, USA). Equal loading was confirmed with β-actin antibodies (Cell Signaling, Danvers, MA, USA) after stripping with Restore™ Western blot stripping buffer (Pierce, Rockford, IL, USA).

### Colony survival assay

Colony survival was performed as described [31]. Cells were plated and exposed to different sources of DNA damage. One hour post-treatment, cells were split into 2,000 cells per 10 cm plate and cell growth assessed after 14 days by staining with 0.5% crystal violet and 25% methanol. The colony number was calculated and plotted as the mean for triplicate samples and presented as percentages relative to the control.

### Apoptosis assay

Apoptosis was assessed by flow cytometric analysis of cells stained with Annexin V-FITC and PI using a Becton Dickinson FACScan (St. Louis, MO, USA) [8,10]. Data analysis using CELLQuest™ software (Becton Dickinson) was performed; numbers of cells positive for Annexin V-FITC, PI, or combinations thereof, were calculated.

### Suppression of IRF5 with siRNA

A modified protocol from Hu *et al*. [10] was used to transfect siRNAs into immortalized non-oncogenic mammary epithelial cells. MCF-12A cells were transfected using Qiagen (Valencia, CA, USA) RNAifect Transfection Reagent once with 5 nM of *IRF5 *pooled siRNAs (Catolog #16708; Ambion, Austin, TX, USA) and harvested 24 h later, or twice (transfected a second time at the 24 h timepoint) and harvested an additional 24 h later. siGenome Lamin A/C Control siRNA (Catalog #D-001050-01-05; Dharmacon, Lafayette, CO, USA) was transfected in a similar manner. Knockdown of IRF5 was determined from Western blots by densitometry analysis of the mean pixel intensity of IRF5 normalized to β-actin.

### 3-Dimensional (3-D) culture in Matrigel and PCR array

3-D culture was performed as described [32]. Cells were spread between two layers of Matrigel (Becton Dickinson) in eight-well chamber slides. Slides were incubated at 37°C in 5% CO_2_/95% air for 10 days. Acini formation was visualized on a Zeiss microscope at 10 × magnification. 3-D colonies were harvested with Cultrex 3D culture Matrix™Cell Harvesting Kit (3448-020-K, Trevigen, Gaithersburg, MD, USA) following the manufacturer's instruction and total RNA isolated with Qiagen RNeasy Plus Mini kit (#74134). Total RNA was converted to cDNA with qScript™ cDNA SuperMix (Quanta BioSciences #84034; Gaithersburg, MD, USA) for PCR array and qPCR analysis. The effect of IRF5 overexpression on 84 known tumor metastases genes was analyzed using the Human Tumor Metastasis RT^2 ^Profiler™ PCR Array (SABiosciences PAHS-028A-2; Frederick, MD, USA) using RT^2 ^SYBR^® ^Green qPCR Master Mixes (SABiosciences, PA-012); qPCR was performed on the ABI 7300 instrument. Raw data were analyzed with SABiosciences online data analysis software. For standard q2PCR, iTaq™SYBR Green Supermix with Rox (Bio-Rad 172-5850; Hercules, CA, USA) was used. Primer sequences for standard qPCR are shown in Additional file 1, Table S1 obtained from the Quantitative PCR Primer Database [33].

### Chemotaxis assay

Chemotaxis assays were performed using 24-well transwell permeable supports (Corning Life Sciences, Lowell, MA, USA) in accordance with the manufacturer's instructions. Briefly, 100 ng/ml human recombinant CXCL12/SDF-1 (R&D Systems, Minneapolis, MN, USA) was added to 600 μl of phenol red-free DMEM medium supplemented with 10% FBS in the lower chamber. A total of 1 × 10^5 ^MDA-MB-231 cells in 100 μl of medium were added to the upper chamber, separated from the lower chamber by a membrane (6.5 mm diameter, 8 μM pore size, polycarbonate membrane). Total cell migration was obtained by calculating cell number in the lower chamber after 6 hr of incubation at 37°C in 5% CO_2_. Three samples were analyzed separately in duplicate, and the data were averaged for statistical analysis.

### Cell surface expression of CXCR4

Cell surface expression of CXCR4 was measured by flow cytometry. MDA-MB-231 cells cultured with and without 100 ng/ml CXCL12 for six hours were stained with PE-conjugated anti-human CXCR4 antibodies or isotype control antibodies (BioLegend, San Diego, CA, USA) in accordance with the manufacturer's specifications. In brief, cells were harvested, washed in PBS, mixed with the appropriate antibody and incubated in the dark for 15 minutes before analysis by flow cytometry. A total of 10,000 events were accumulated for each analysis; samples were analyzed in triplicate.

### CXCR4 promoter reporter assay

A total of 1 × 10^6 ^MDA-MB-231/pBabe or MDA-MB-231/pBIRF5 cells were plated in 96-well format in triplicate four hours before transfection (SuperFect Transfection Reagent, Qiagen) with pGL3 empty vector control plasmid or the *CXCR4 *luciferase promoter reporter pGL3-*CXCR4*/3B/4-1(5'Δ3) (-191 to +88) [34] from Dr. Nelson L. Michael (Walter Reed Army Institute of Research). In all wells, 40 ng of thymidine kinase driven Renilla luciferase reporter gene (Promega, Madison, WI, USA) was co-transfected to normalize for transfection efficiency. After 24 h of transfection, fresh media was added to cells with or without 100 ng/ml CXCL12 for 4 h. Post-stimulation, cell lysates were prepared, and reporter gene activity was measured using the Dual luciferase assay system (Promega) [5]. Data are expressed as the mean relative stimulation ± S.D.

### In vivo tumorigenicity assay

Four- to six-week ovariectomized, Ncr *nu/nu *mice (*n *= 18 per group (Charles Rivers Laboratory, Wilmington, MA, USA) were supplemented with 17 β-estradiol pellets (0.72 mg/pellet; Innovative Research of America, Sarasota, FL, USA) and used to determine the tumorigenicity of MCF-7 pooled stable transfectants [26]. A total of 1 × 10^6 ^control (MCF-7/pBabe) or MCF-7/pBIRF5 cells were inoculated into opposite thoracic mammary fat pads. Ncr *nu/nu *mice (*n *= 15 per group) were also used for MDA-MB-231 pooled stable transfectants. A total of 2 × 10^6 ^control (MDA-MB-231/pBabe) or MDA-MB-231/pBIRF5 cells were inoculated into upright mammary fat pads. The primary endpoint was the incidence of proliferating tumors; secondary was tumor size. Tumor areas were estimated from the product of the two longest perpendicular measurements with a caliper. All *in vivo *studies were conducted in accordance with UMDNJ New Jersey Medical School Animal Care and Use Committee approved protocols.

### Statistical analyses

Data are presented as mean ± SD of data obtained from three or four independent experiments performed in duplicate. Representative experiments of multiple experiments are depicted in some figures. Comparisons between values were analyzed by the Student's *t*-test. Differences were considered significant at *P*-values ≤ 0.05. Statistical analyses were performed using Prism 4.0 (GraphPad Software, San Diego, CA, USA). Cumulative incidences of proliferating tumors in each experimental group were visualized by the Kaplan-Meier method and compared by the log rank test.

## Results

### Loss of IRF expression in human breast tumor tissues

**Figure 1:**
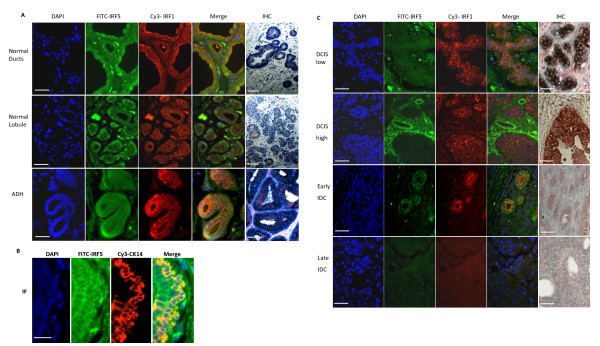
**Dysregulated IRF expression in patients with ductal carcinoma**. **A**. Normal and ADH breast tissue specimens were stained by IF or IHC. Antibodies recognizing IRF5 (FITC), IRF1 (Cy3) and DAPI for the nucleus were used for IF. For IHC, tissues were stained for IRF1 with DAB (brownish-red), IRF5 with BAP (blue), and nucleus with Fast Red mounting buffer. **B**. Same as in (A), except tissue samples from patients with ADH were stained by IF with IRF5 (FITC) and CK14 (Cy3) in order to confirm expression of IRF5 in myoepithelial cells. **C**. Same as in (A), except tissues from patients with DCIS and IDC were examined. Representative pictures of low grade and high grade DCIS are shown illustrating distinct differences between IRF1 and IRF5 expression. Images were taken on a Zeiss Axiovert Apotome microscope at 20 × or 40 × magnification. Scale bars are 50 μm.

IRF1 and IRF5 expression were examined in FFPE specimens from patients with different stages of breast cancer by IF and IHC. Normal ducts gave diffuse cytoplasmic and nuclear staining for IRF1, as shown by the purple color in the merged IF panel, as well as IHC, that was consistent with previous findings [25]. In contrast, IRF5 staining appeared primarily diffuse cytoplasmic. Staining of normal lobules (by IF and IHC) revealed that IRF1 was highly expressed in luminal epithelial cells lining the duct and IRF5, while also detected at low levels in these cells, was more focused and pronounced in ductal myoepithelial cells (MECs); results from IF of normal ducts and ADH support the differential cellular expression of IRF1 and IRF5 (Figure 1A). Co-staining with cytokeratin 14 (CK14) confirmed expression of IRF5 in MECs (Figure 1B).

**Figure 2:**
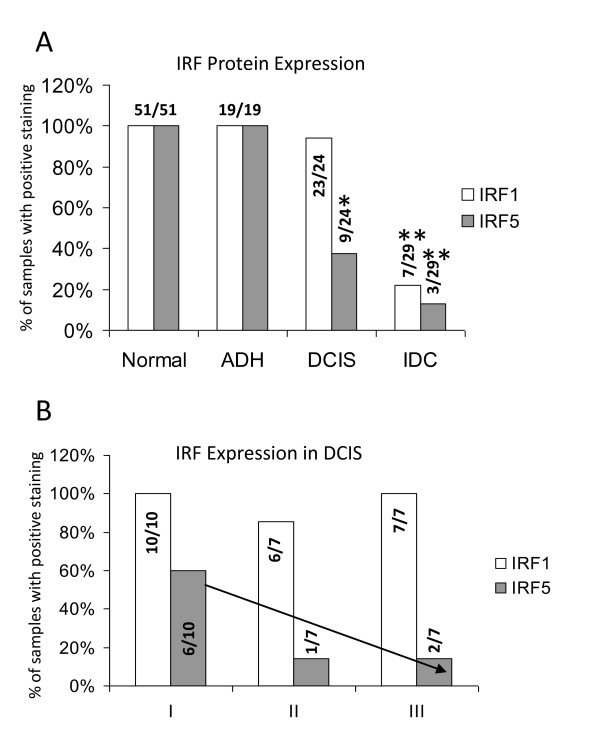
**Summary of IRF expression in breast tissue specimens**. **A**. Percent of samples with positive staining is shown on y-axis, tissue and disease type are shown on x-axis. Number of samples positive for IRF1 or IRF5 is shown over total number of patient samples examined in each group. Statistical significance was determined by comparing the number of positive-stained samples in each disease type to positive-stained samples in normal tissues; * denotes *P *< 0.05, **P < 0.001. **B**. Same as in A, except expression in different stages of DCIS is shown.

Of the 19 patients with ADH, 100% showed positive staining for IRF1 and IRF5 (Figures 1A and 2A). Most DCIS breast cancers retained expression of IRF1 in tumor tissue with 23 out of 24 staining positive, whereas IRF5 expression was significantly reduced with only 9 out of 24 staining positive (Figures 1C and 2A). A distinct trend was observed when examining IRF5 expression in low and high grades of DCIS marking a grade-dependent decrease in expression (Figure 2B). Of the 29 patients with IDC, only 7 retained IRF1 expression and 3 had IRF5 (Figure 1C, 2A). Throughout our analysis of stained slides, reviewers noted that IRF5 and not IRF1 expression was often detected in the surrounding stroma of DCIS and IDC patients (Figure 1).

Review of pathology data, including grade as scored by the original clinical pathologist, estrogen (ER), progesterone (PR), and Her2 receptor status, was performed by Dr. Hameed, a licensed pathologist, under an approved NJMS IRB. Given the small sample size, it was difficult to make statistical correlations between receptor and IRF expression; however, data at present suggest that loss of IRF5 expression correlates with ER/PR(-) breast cancers in 82 to 90% of samples. Loss of Her2/neu expression did not correlate with IRF5 expression. Evaluation of IRF1 expression in relation to tumor characteristics gave no correlations [25].

### IRF5 modulates cell growth and response to DNA damage

**Figure 3:**
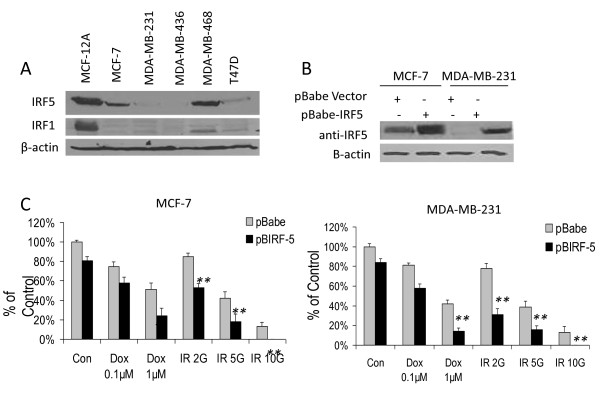
**Overexpression of IRF5 in MCF-7 and MDA-MB-231 cells sensitizes them to DNA damage-induced growth inhibition**. **A**. Endogenous IRF expression was analyzed by Western blot in transformed mammary epithelial cell lines. Levels of β-actin are shown as loading controls. **B**. Western blot analysis of stable cell lines generated to overexpress retroviral pBIRF5. **C**. Cell survival was measured in MCF-7 and MDA-MB-231 pBabe cell lines by colony formation assay before and after treatment. Cells were treated with 0.1 or 1 μM Doxorubicin (Dox) or 2, 5 and 10 Gy γ-IR. The number of colonies is plotted on the y-axis as percent of control; 100% represents the number of colonies in empty pBabe control lines. Data are expressed as mean ± SD of three independent experiments performed in duplicate. Statistical significance was determined by comparing the difference between colonies in pBabe versus pBIRF5 cell lines after each treatment; * denotes *P *< 0.05, ** *P *< 0.001.

We next examined IRF1 and IRF5 protein expression in immortalized tumorigenic mammary epithelial cells and non-oncogenic mammary epithelial cell lines to confirm findings in primary tissues. IRF1 and IRF5 levels were consistently reduced in breast cancer cell lines compared to non-oncogenic mammary epithelial MCF-12A cells (Figure 3A); similar findings were made at the transcript level by Q-PCR (data not shown or Additional file 2). To address the functional consequence of this loss, we examined the reciprocal effect of overexpression in cell lines that had little or no endogenous expression. MCF-7 (ER(+), low invasive) and MDA-MB-231 (ER(-), highly invasive and tumorigenic) cells were generated to stably overexpress IRF5 by retroviral infection (Figure 3B). Control lines expressing empty vector pBabe were generated at the same time and confirmed to grow and respond identical to parental cells (data not shown). The colony formation assay was used to determine the effect of IRF5 on cell growth. IRF5 reduced colony formation by approximately 20% as compared to unstimulated empty vector controls (Figure 3C). These data are consistent with earlier findings of IRF5 function in lymphoma, lung and colon cancer cells [7,8,23]. Depending on cell type, IRF5 can have little effect on growth or apoptosis in unstimulated cells [7,8]. IRF5 generally requires activation and nuclear localization for its biological function [7,8]; however, transient or stable overexpression has been demonstrated to push IRF5 into the nucleus resulting in low but significant growth inhibition [3,4,7,8,23]. To determine whether IRF5 function in breast cancer was dependent on DNA damage, cells were treated with Doxorubicin (Dox) or exposed to γ-irradiation (IR). IRF5 sensitized cells to DNA damage-induced growth inhibition in a similar dose-dependent manner independent of the source of damage both in 2D culture (data not shown) and in colony formation assay (Figure 3C). These data support that IRF5 enhances DNA damage-induced growth inhibition.

**Figure 4:**
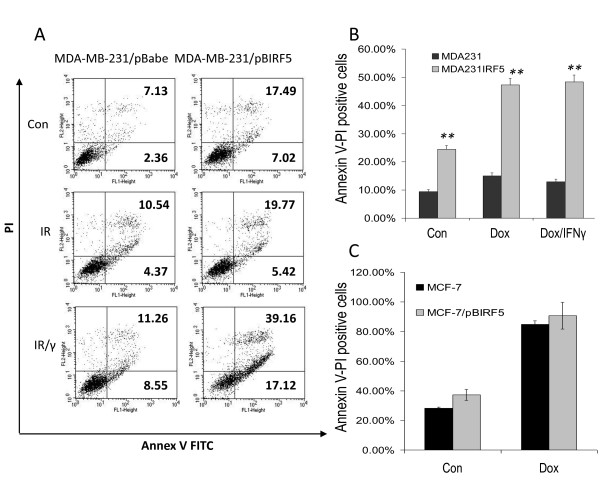
**Overexpression of IRF5 in MDA-MB-231 cells sensitizes them to IR-induced apoptosis**. **A**. MDA-MB-231 cells were exposed to 5 Gy IR or the same dose plus IFN-γ (IR/γ) for 24 h. Percent of cells undergoing apoptosis was measured by FACS analysis of Annexin V-FITC (x-axis) and PI (y-axis) double-staining. Percent of Annexin V-FITC stained positive cells is shown in the upper and lower right-hand quadrants. Representative histogram plots from three independent experiments performed in duplicate are shown. **B**. Same as in (A), except cells were treated with 1 μM Dox or the same dose plus IFN-γ (Dox/γ) for five hours. Percent of Annexin V-FITC-stained positive cells compared to control is plotted on y-axis. Data are expressed as mean ± SD of three independent experiments performed in duplicate. Statistical significance was determined by comparing the difference between pBabe and pBIRF5 cells lines after each treatment; ** denotes *P *< 0.001. **C**. Same as in (B), except MCF-7 cells were treated with Dox.

To determine whether the observed growth inhibition was due to IRF5-mediated apoptosis or necrosis, we measured AnnV-FITC and PI-double staining by flow cytometry. Overexpression in untreated MDA-MB-231 cells (MDA-MB-231/pBIRF5) induced apoptosis approximately 2.5-fold over empty vector control cells (Figure 4A). No significant difference in total AnnV-FITC positive-stained cells was observed between untreated and IR-treated cells; yet, when compared to IR-treated empty vector control cells, IRF5 provided an approximately 40% increase in positive-stained cells. Combinations of IR/IFN-γ with IRF5 provided a synergistic induction of apoptosis. Similarly, MDA-MB-231/pBIRF5 cells were sensitized to Dox-induced apoptosis while no synergistic or additive effects were observed with IFN-γ (Figure 4B). Overexpression of IRF5 in MCF-7 cells (MCF-7/pBIRF5) had no significant effect on IR- or Dox-induced apoptosis; cells were also resistant to combinations with IFN-γ (Figure 4C and data not shown). Previous data from our lab showed a synergistic effect of DNA damage and type I (α and β) or II (γ) IFNs [7,8]. IFN-γ on its own has very little effect on MCF-7 or MDA-MB-231 cells [35] (data not shown). Whether the differential effects observed between these two cell lines were due to ER status or other genetic/biological differences is unclear at this time. Nonetheless, data in Figures 3 and 4 indicate that IRF5-mediated growth inhibition is in part independent of its ability to mediate apoptosis.

### IRF5 knock-down in immortalized non-oncogenic mammary epithelial cells confers protection from DNA damage-induced growth inhibition and apoptosis

**Figure 5:**
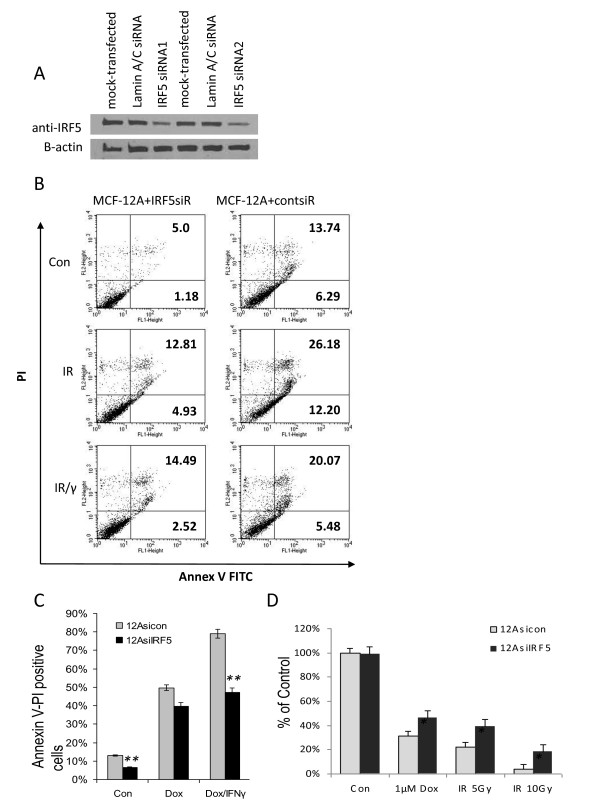
**Down-regulation of IRF5 protein expression by siRNAs alters sensitivity to DNA damage**. **A**. MCF-12A cells were incubated with transfection reagent alone (mock-transfected), control Lamin A/C siRNAs or 5 nM *IRF5 *siRNAs once (IRF5 siRNA1) or twice (IRF5 siRNA2), as described in the Materials and methods. Western blot analysis shows > 70% reduction of endogenous IRF5 proteins after normalization to β-actin levels. **B**. Cells were exposed to 5 Gy IR or the same dose plus IFN-γ (IR/γ) for 24 h. Percent of Annexin V-FITC stained positive cells is shown in the upper and lower right-hand quadrants. Representative histogram plots from three independent experiments performed in duplicate are shown. **C**. Same as in B, except cells were exposed to 1 μM Dox or Dox and IFN-γ for five hours. Percent of Annexin V-FITC stained positive cells compared to control is plotted on y-axis. Data are expressed as mean ± SD of three independent experiments performed in duplicate. Statistical significance was determined by comparing the difference between cells transfected with Lamin A/C siRNAs (12Asicon) and IRF5 siRNAs (12AsiIRF5) after each treatment; ** denotes *P *< 0.001. **D**. Cells were treated with the indicated doses of Dox or IR after siRNA transfection. Number of colonies is plotted on y-axis as percent of control. A total of 100% represents the number of colonies in control untreated 12Asicon cells. Data are expressed as mean ± SD of three independent experiments performed in duplicate. Statistical significance was determined by comparing the difference between colonies in 12Asicon versus 12AsiIRF5 cells after each treatment; * denotes *P *< 0.05.

The fact that IRF5 is well expressed in immortalized non-oncogenic mammary epithelial cells compared to breast cancer cells and tumor tissues is consistent with the concept that IRF5 is a tumor suppressor protein. Overexpression studies confirm a role for IRF5 in cell growth and response to DNA damage (Figures 3 and 4). We next examined the direct consequences of loss of IRF5 expression on the DNA damage response in immortalized non-oncogenic mammary epithelial MCF-12A cells. Western blot data in Figure 5A show > 70% reduction of IRF5 proteins, as determined by densitometry analysis, after single (IRF5 siRNA1) or double (IRF5 siRNA2) transfections with a pool of three targeted *IRF5 *duplexes (see Materials and methods). The double transfection (IRF5 siRNA2) protocol, harvested at 48 h post-transfection, was used in all further experiments since it gave approximately 80% reduction. No differences in IRF5 expression or cellular growth were observed in cells mock transfected or transfected with control siRNAs (Figure 5A and data not shown). Knockdown of IRF5 in MCF-12A cells resulted in significant protection (> 50%) from normal or spontaneous apoptosis. Significant reductions in IR-induced apoptosis were also observed, whereas little protection was offered in response to Dox (Figure 5B, C). Significant protection from Dox/IFN-γ treatment was observed in cells transfected with *IRF5 *siRNAs. Loss of IRF5 expression also protected cells from DNA damage-induced growth inhibition (Figure 5D). These data implicate IRF5 as a critical regulator of *in vitro *mammary epithelial cell growth and response to DNA damage.

### IRF5 modulates in vivo/in vitro tumor cell growth and metastasis/invasion by regulating CXCR4 expression

**Figure 6:**
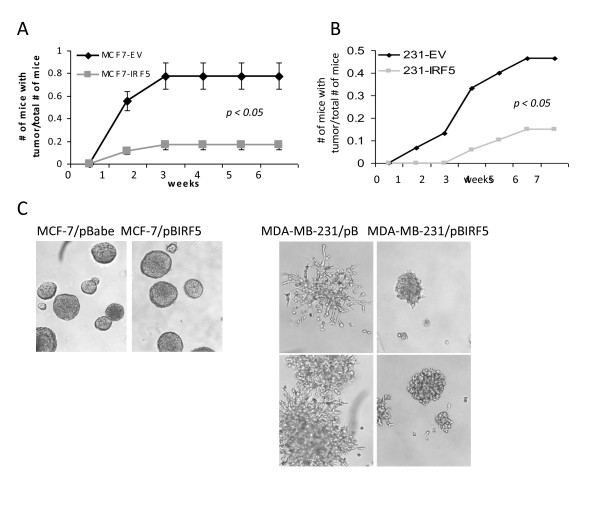
**IRF5 inhibits *in vivo *tumor formation and *in vitro *metastasis/invasion**. **A**. MCF-7/pBIRF5 (MCF7-IRF5) and MCF-7/pBabe (MCF7-EV) control cells were inoculated into NCr *nu/nu *mice. The number of mice with tumors over the total number of mice is shown. **B**. Same as in (A), except 3 × 10^6 ^MDA-MB-231 control cells (231-EV) or MDA-MB-231 IRF5 overexpressing cells (231-IRF5) were inoculated into NCr *nu/nu *mice and monitored over seven weeks. **C**. Growth of MCF-7 and MDA-MB-231 cells were examined by 3-D culture. An equal number of cells were plated and pictures taken 10 days later at 10 × magnification.

To determine directly whether IRF5 could act as a tumor suppressor *in vivo*, MCF-7/pBabe and MCF-7/pBIRF5 cells were inoculated into NCr *nu/nu *mice. The cumulative incidence of proliferating tumors was significantly lower for MCF-7/pBIRF5 mice compared with controls and the few tumors that formed (in 3 out of 18 mice) were significantly smaller than control (Figure 6A and data not shown). Similar findings were made after injection with MDA-MB-231/pBabe and MDA-MB-231/pBIRF5 cells (Figure 6B).

Intracardiac or intravenous injection of MDA-MB-231 cells generates a rapid experimental model of tumor metastasis. While injection of MDA-MB-231 cells into mammary fat pads also models tumor metastasis, low incidence of primary tumor formation (50% of mice) and later metastases (approximately 20%) was observed. Nonetheless, mice injected with MDA-MB-231/pBIRF5 cells that generated primary tumors showed no metastases. This, combined with *in vitro *data suggesting other mechanisms of IRF5-mediated growth inhibition, led us to examine the effect of IRF5 on tumor cell metastasis/invasion. By 3-D cell culture, IRF5 overexpression in MDA-MB-231 but not MCF-7 cells inhibited acini outgrowth (Figure 6C). The lack of response in MCF-7 cells to IRF5 was expected given the low metastatic potential of these cells. To determine how IRF5 inhibited outgrowth, we examined expression of genes contained in a pre-designed Human Tumor Metastasis PCR array (see Materials and methods). Differences were observed between pBabe and pBIRF5 cell lines; genes showing differential regulation included *CTSK*, *CXCR4*, *HGF*, *ITGA7*, *MMP10*, and *RORB *(Table 1). Independent analysis of these genes confirmed a significant down-regulation of *CXCR4 *by IRF5 (5- to -6-fold decrease; *P *< 0.05) (Additional file 3).

**Table 1:** Genes differentially regulated by IRF5 in MDA-MB-231 cells.

Gene	Function	Expression in pBIRF5 vs. pBabe
*CTSK^a^*	Cathepsin K	Decreased
*CXCR4^b^*	Receptor for CXCL12/SDF-1	Decreased
*HGF^a^*	Hepatocyte growth factor	Decreased
*ITGA7^b^*	Integrinα7	Decreased
*MMP10^a^*	Matrix metalloproteinase 10	Decreased
*MMP3^b^*	Matrix metalloproteinase 3	Increased
*RORB^b^*	Retinoid-related orphan receptor β	Increased

^a^denotes genes with ≥ 2- to 3.99-fold change; ^b^genes with ≥ 4- to 6.99-fold change. Gene expression was compared between MDA-MB-231/pBabe and MDA-MB-231/pBIRF5.

**Figure 7:**
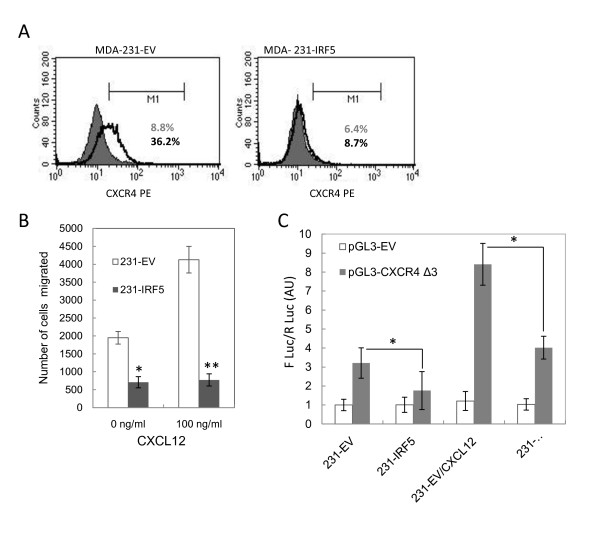
**IRF5 reduces CXCR4 cell surface expression and SDF-1/CXCL12-dependent chemotaxis of MDA-MB-231 cells**. **A**. CXCR4 expression (grey line) in unstimulated cells, shown superimposed on the isotype control (grey shaded area), and CXCR4 expression (black line) after stimulation, was measured by flow cytometry. MDA-MB-231 cells (pBabe and pBIRF5) were treated with the CXCR4 ligand SDF-1 for six hours and CXCR4 expression measured. IRF5 expressing cells show no significant expression of CXCR4. M1, Marker 1. Representative histogram plots from three independent experiments performed in duplicate are shown. **B**. Cells overexpressing IRF5 are incapable of SDF-1-induced migration when compared to empty vector (EV pBabe) control cells. Data are expressed as mean ± SD of three independent experiments performed in duplicate. Statistical significance was determined by comparing the difference in number of cells migrated between pBabe and pBIRF5 cells; * denotes *P <*0.02, **P <0.005. **C**. *CXCR4 *promoter reporter activity was analyzed by Dual Luciferase assay. MDA-231-pBabe and MDA-231-pBIRF5 were transfected with pGL3 empty vector or pGL3*CXCR4*5'Δ3 promoter and mock-treated with PBS or 100 ng/ml CXCL12. Data are expressed as the mean relative stimulation ± SD from three independent experiments performed in triplicate. Statistical significance was determined by comparing the difference in promoter activity between pBabe and pBIRF5 expressing cells; * denotes *P *< 0.05.

To ensure that there is a correlation between *CXCR4 *mRNA and cell surface expression of the CXCR4 protein, we performed flow cytometric analysis. Cell surface expression of CXCR4 in unstimulated MDA-MB-231 cells is very low and no significant difference was observed in basal CXCR4 expression between MDA-MB-231/pBabe (8.8%) and MDA-MB-231/pBIRF5 (6.4%) cells (Figure 7A, shown by a grey line superimposed on isotype control peak). Therefore, to ensure an accurate measure of CXCR4 cell surface expression on MDA-MB-231/pBabe and MDA-MB-231/pBIRF5 cells, cells were incubated for six hours with the CXCR4 ligand SDF-1/CXCL12 to upregulate CXCR4 expression. Data in Figure 7A clearly demonstrate the inability of MDA-MB-231/pBIRF5 cells to express surface CXCR4 (shown by black line). By chemotaxis assay, we show that MDA-MB-231/pBIRF5 cells, as compared to MDA-MB-231/pBabe cells, are incapable of migrating in response to SDF-1 (Figure 7B). Furthermore, in the absence of SDF-1/CXCL12, basal migration was significantly inhibited supporting our findings in 3-D culture.

Since these data suggested that IRF5 may contribute to the regulation of CXCR4 in breast cancer cells, we performed a computer-based analysis of the human *CXCR4 *gene promoter with MatInspector [36]; two IRF binding elements (IRF-E) were identified. *CXCR4 *promoter reporter assays were subsequently performed in MDA-MB-231/pBabe and MDA-MB-231/pBIRF5 cells. Data in Figure 7C indicate basal transactivation of the *CXCR4 *promoter that was significantly down-regulated in cells expressing IRF5. Similar findings were made in MCF-7 cells transfected with Flag-tagged IRF5 (data not shown). Stimulation of MDA-MB-231/pBabe cells with CXCL12/SDF-1 ligand greatly enhanced reporter activity, while stable MDA-MB-231/pBIRF5 cells yielded a significant decrease in ligand-induced transactivation. These data confirm the negative regulation of CXCR4 expression by IRF5 in breast cancer cells.

Further support for IRF5 in regulating tumor metastasis was obtained by examining IRF5 expression in clinical metastatic lymph node tissues from IDC patients. As expected, all samples examined were negative for IRF5 expression, except one that showed very low levels (Additional file 4), supporting a correlation between loss of IRF5 expression and tumor metastases.

## Discussion

Results presented here provide the first clear support of IRF5 tumor suppressor function and identify a new role for IRF5 in tumor cell invasion/metastasis. We demonstrated that loss of IRF5 expression correlated with advanced stages of breast cancer and invasion/metastasis. Loss of IRF5 preceded that of IRF1, but loss of IRF5 expression was not a prerequisite for IRF1 and IRF5 overexpression did not affect IRF1 levels (Figure 3A and data not shown). IRF1 was used as a comparative control given its known expression and function in breast cancer [25]. The differential reactivity of the IRF1 and IRF5 antibodies by IF and IHC, as well as by Western blot showing they bind to discrete molecular weight bands (IRF1 approximately 48 kDa and IRF5 62 kDa), support their specificity; in addition, the same IRF1 antibody used in the manuscript by Doherty *et al*. [25] to examine IRF1 expression in FFPE samples was used in this study. Two distinct IRF5 antibodies, one from Novus Biologicals and the other from Cell Signaling, were tested and gave identical results by IF, IHC and Western blot analysis of IRF5 expression in immortalized transformed and untransformed cell lines (data not shown). Together, these data document both the specificity and non-cross-reactivity of anti-IRF1 and anti-IRF5 antibodies.

Although we found that IRF1 and IRF5 were similarly expressed in normal breast tissue and patients with ADH or IDC, significant differences were observed in DCIS suggesting the unique utilization of these two biomarkers for diagnosis and prognosis. Another important distinction between these two transcription factors was in cellular expression; IRF5 was predominantly expressed in MECs (Figure 1A, B). IRF5 was also detected in non-MECs and the surrounding stroma of early DCIS, late DCIS and IDC patients (Figure 1C). These data support distinct functions for IRF1 and IRF5 in breast tumorigenesis. MECs play a critical role in mammary gland development and loss of myoepithelial function is almost universally associated with breast cancer [37]. MECs are localized between luminal epithelial cells and the stroma, which ideally position them to communicate with both compartments. They suppress tumor growth and invasion [38] and degradation of the MEC layer and basement membrane is an absolute prerequisite for breast cancer invasion and metastasis [39]. Mounting evidence also demonstrates the importance of surrounding stroma in tumor promotion [40]. Recent data from Eguchi *et al*. support a role for IRF5 in the fatty stroma [41]. Additional experiments are necessary to determine the exact expression and function of IRF5 in tumor versus non-tumor MECs, stromal cells and non-MECs. Significant differences in gene expression have been observed between normal MECs and tumor MECs [42,43]. Given the known function(s) of IRF5 in regulating proinflammatory cytokine/chemokine expression [3,4,6], combined with its cellular expression in breast tissue and high expression in infiltrating leukocytes in the tumor stroma of IDC patients (Additional file 5), suggest that IRF5 may play an important role in breast cancer invasion. Indeed, the van't Veer cohort placed IRF5 in a dominant gene cluster associated with lymphocytic infiltration and progressive disease [44]. Furthermore, IRF5 is part of a 28-gene signature for predicting breast cancer recurrent and metastatic potential [45]. Based on data presented here, we propose a two-fold function for IRF5 that is cell type-specific and lends support to the 'release' model of breast cancer invasion where phenotypic changes in MECs (loss of IRF5 expression), in coordination with the infiltration and influence of inflammatory cells (high levels of IRF5 expression), lead to the breakdown of ducts and release and invasion of tumor epithelial cells [46].

Clinical data from tissue specimens combined with expression analyses and 3-D cultures provide the first clues that IRF5 may be involved in regulating tumor metastases, where loss of IRF5 enhances metastatic potential. A cursory review of the literature indicates that this function is unique to IRF5 and not IRF1. The molecular mechanism by which IRF5 inhibits invasion/metastasis is not yet clear but likely involves the dysregulation of genes, such as *CXCR4*. *CXCR4*, the receptor for chemokine CXCL12/SDF-1, was significantly down-regulated at both the transcript and protein level by IRF5 overexpression, and IRF5 inhibited promoter reporter activity (Figure 7A, C and Additional file 3). CXCR4 is an important factor in the migration, invasiveness and proliferation of breast cancer cells and silencing of *CXCR4 *blocks breast metastasis [47,48]. Increased expression of CXCR4 in primary breast tumors has been associated with developing bone metastases [49].

Further studies will be necessary to address the question of how or why IRF5 expression is altered in different stages of human breast cancer. Results from Q-PCR analysis of *IRF5 *transcript expression (Additional file 2) support the presence of *IRF5 *transcripts in cell lines that lack detectable IRF5 proteins, that is, MDA-MB-231 and T47D cells, yet the overall trend in IRF5 transcript and protein levels correlated. The *IRF5 *promoter does contain a large CpG rich island [13] suggesting that it may be susceptible to silencing by hypermethylation; yet, when MDA-MB-231, MDA-MB-436 and T47D cell lines were treated with 5-aza-2'-deoxycytidine and *IRF5 *expression analyzed by RT-PCR, no change in transcript levels was detected (data not shown). It has recently been demonstrated that the *IRF5 *promoter is frequently hypermethylated in hepatocellular carcinoma tissue samples [50]. A similar study in immortalized cell lines from patients with Li-Fraumeni syndrome that had decreased *IRF5 *expression showed no detectable methylation of CpG islands in the *IRF5 *promoter [51]. More recently, a single point mutation in the *IRF5 *gene was identified in peripheral blood from patients with adult T-cell leukemia/lymphoma (ATL) and chronic lymphocytic leukemia (CLL) that altered the function of wild-type IRF5 [52]. Together, these data suggest that multiple mechanisms may exist that regulate IRF5 expression and function in cancer.

## Conclusions

Altogether, data presented here support a differential role for IRF1 and IRF5 in breast tumorigenesis warranting further investigation regarding prognostic and therapeutic implications. While both are important, loss of each of these factors may play distinct roles in the conversion of DCIS to IDC and the later metastasis of primary tumors.

## Abbreviations

ADH: atypical ductal hyperplasia; ATL: adult T-cell leukemia/lymphoma; CLL: chronic lymphocytic leukemia; DCIS: ductal carcinoma in situ; Dox: doxorubicin; ER: estrogen; FBS: fetal bovine serum; FFPE: formalin-fixed paraffin-embedded; HRP: horse radish peroxidase; IDC: invasive ductal carcinoma; IL: interleukin; IF: immunofluorescence; IFN: interferon; IHC: immunohistochemistry; IR: γ-irradiation; IRB: institutional review board; IRF: interferon regulatory factor; MEC: myoepithelial cells; MEF: mouse embryonic fibroblast; PI: propidium iodide; PR: progesterone; siRNA: silencing RNA; stromal derived factor-1: SDF-1/CXCL12; TLR: toll-like receptor.

## Competing interests

The authors declare that they have no competing interests.

## Authors' contributions

XB contributed to all aspects of the study including the design, statistical analysis and manuscript preparation, as well as carried out all *in vitro *and *in vivo *scientific assays. MH and NM analyzed H&E sections of FFPE tissue specimens, and graded and selected cases to be examined. EMP performed QPCR assays and JA participated in the staining of FFPE tissue specimens. BJB conceived and designed the study, performed statistical analyses, coordinated the requisition of FFPE tissue specimens and drafted the manuscript. All authors read and approved the final manuscript.

## Supplementary Material

Additional file 1 **qPCR primers for PCR array confirmation**. List of primers and sequences that were used for qPCR analysis of genes identified by PCR array. Click here for file

Additional file 2 ***IRF5 *transcript levels are decreased in immortalized breast cancer cell lines as compared to immortalized non-oncogenic mammary epithelial cells**. Results from qPCR of *IRF5 *expression in immortalized mammary cell lines. Click here for file

Additional file 3 ***CXCR4 *transcript levels are decreased in MDA-MB-231/pBIRF5 cells**. Independent analysis of genes identified from PCR array by qPCR. Click here for file

Additional file 4 **IRF1 and IRF5 expression are absent in lymph node mets**. Lymph node metastases from IDC patients were stained for IRF1 and IRF5 expression and analyzed by immunofluorescence. Click here for file

Additional file 5 **IRF5 is highly expressed in immune/inflammatory cells surrounding normal ducts of IDC patients**. IRF1 and IRF5 expression levels were examined by immunofluorescence. Click here for file
