# Supplementary material for: Loss of interferon regulatory factor 5 (IRF5) expression in human ductal carcinoma correlates with disease stage and contributes to metastasis
Source: Breast Cancer Res. 2011 Nov 4;13(6):R111. doi: 10.1186/bcr3053 (PMC3326553; doi:10.1186/bcr3053)
Supplement: Additional file 1 — qPCR primers for PCR array confirmation. List of primers and sequences that were used for qPCR analysis of genes identified by PCR array. [file bcr3053-S1.PDF]

Oligo Name	Sequence 5' to 3'
hCCL2f	CATTGTGGCCAAGGAGATCTG
hCCL2R	CTTCGGAGTTTGGGTTTGCTT
hCCL5f	TACCATGAAGGTCTCCGC
hCCL5R	GACAAAGACGACTGCTGG
hCXCL8f	GAACTGAGAGTGATTGAGAGTGGA
hCXCL8R	CTCTTCAAAAACTTCTCCACAACC
hCXCL12f	AATTCTCAACACTCCAAACTGTGC
hCXCL12R	TGCACACTTGTCTGTTGTTGTTC
hCXCR4f	CCTATGCAAGGCAGTCCATGT
hCXCR4R	GGTAGCGGTCCAGACTGATGA
hCXCL3f	TGGTCACTGAACTGCGCT
hCXCL3R	ATGCGGGGTTGAGACAAG
hMMP2f	TGATCTTGACCAGAATACCATCGA
hMMP2R	GGCTTGCGAGGGAAGAAGTT
hMMP3f	TGGCATTCAGTCCCTCTATGG
hMMP3R	AGGACAAAGCAGGATCACAGTT
hMMP9f	TGGGGGGCAACTCGGC
hMMP9R	GGAATGATCTAAGCCCAG
hTIMP3f	CCAGGACGCCTTCTGCAAC
hTIMP3R	CCTCCTTTACCAGCTTCTTCCC
hβ-actinf	ATAGCACAGCCTGGATAGCAACGTAC
hβ-actinR	CACCTTCTACAATGAGCTGCGTGTG

Supplementary Table 1
