# Supplementary figures and images for: Loss of interferon regulatory factor 5 (IRF5) expression in human ductal carcinoma correlates with disease stage and contributes to metastasis
Source: Breast Cancer Res. 2011 Nov 4;13(6):R111. doi: 10.1186/bcr3053 (PMC3326553; doi:10.1186/bcr3053)

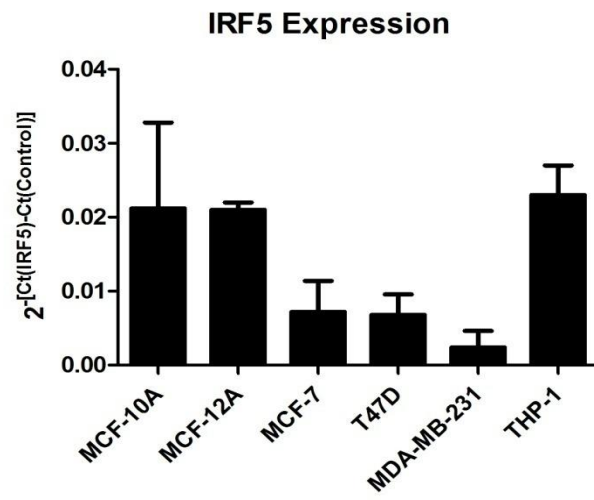

Supplement: Additional file 2 — IRF5 transcript levels are decreased in immortalized breast cancer cell lines as compared to immortalized non-oncogenic mammary epithelial cells. Results from qPCR of IRF5 expression in immortalized mammary cell lines. [file bcr3053-S2.PDF]

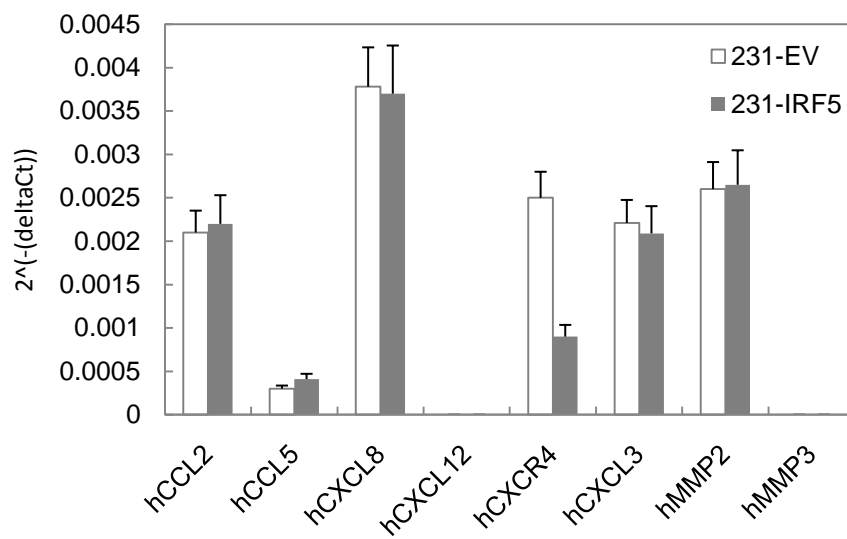

Supplement: Additional file 3 — CXCR4 transcript levels are decreased in MDA-MB-231/pBIRF5 cells. Independent analysis of genes identified from PCR array by qPCR. [file bcr3053-S3.PDF]

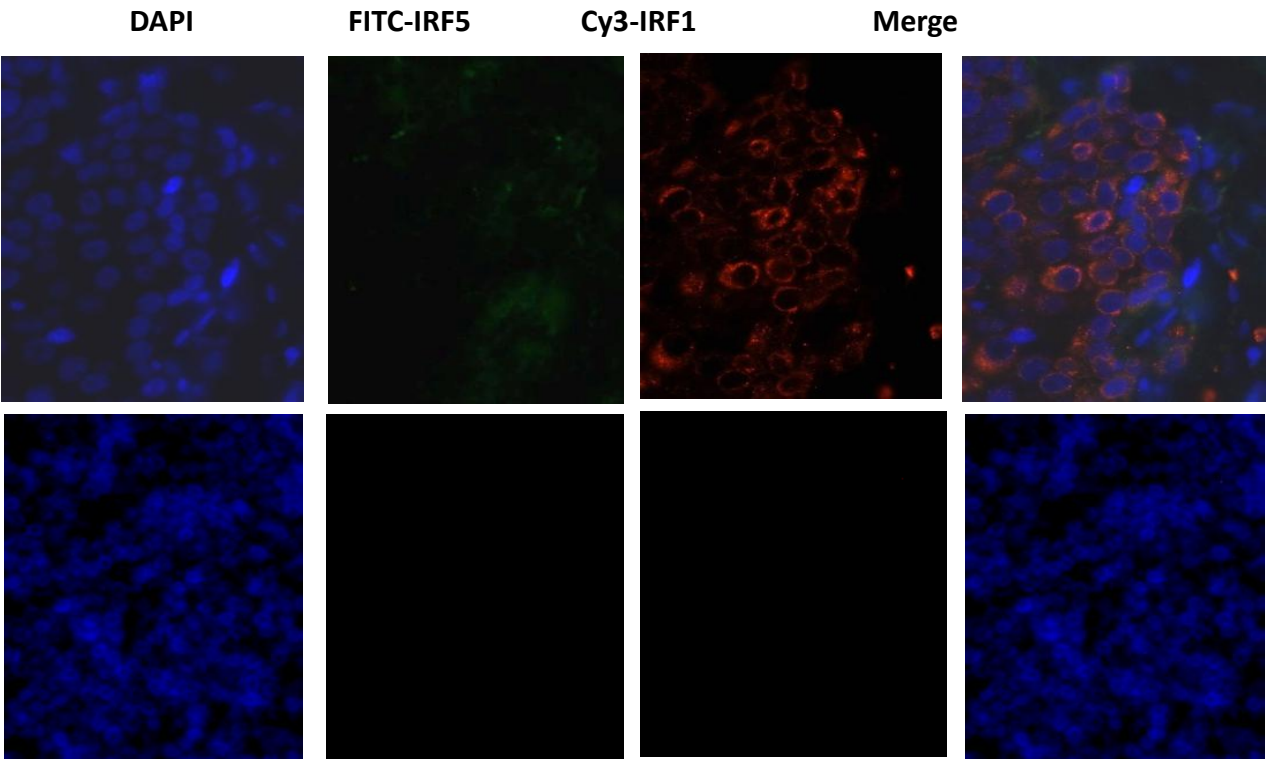

|      |           |
|------|-----------|
|      | LN<br>Met |
| IRF1 | 2/11      |
| IRF5 | 1/11      |

Supplement: Additional file 4 — IRF1 and IRF5 expression are absent in lymph node mets. Lymph node metastases from IDC patients were stained for IRF1 and IRF5 expression and analyzed by immunofluorescence. [file bcr3053-S4.PDF]

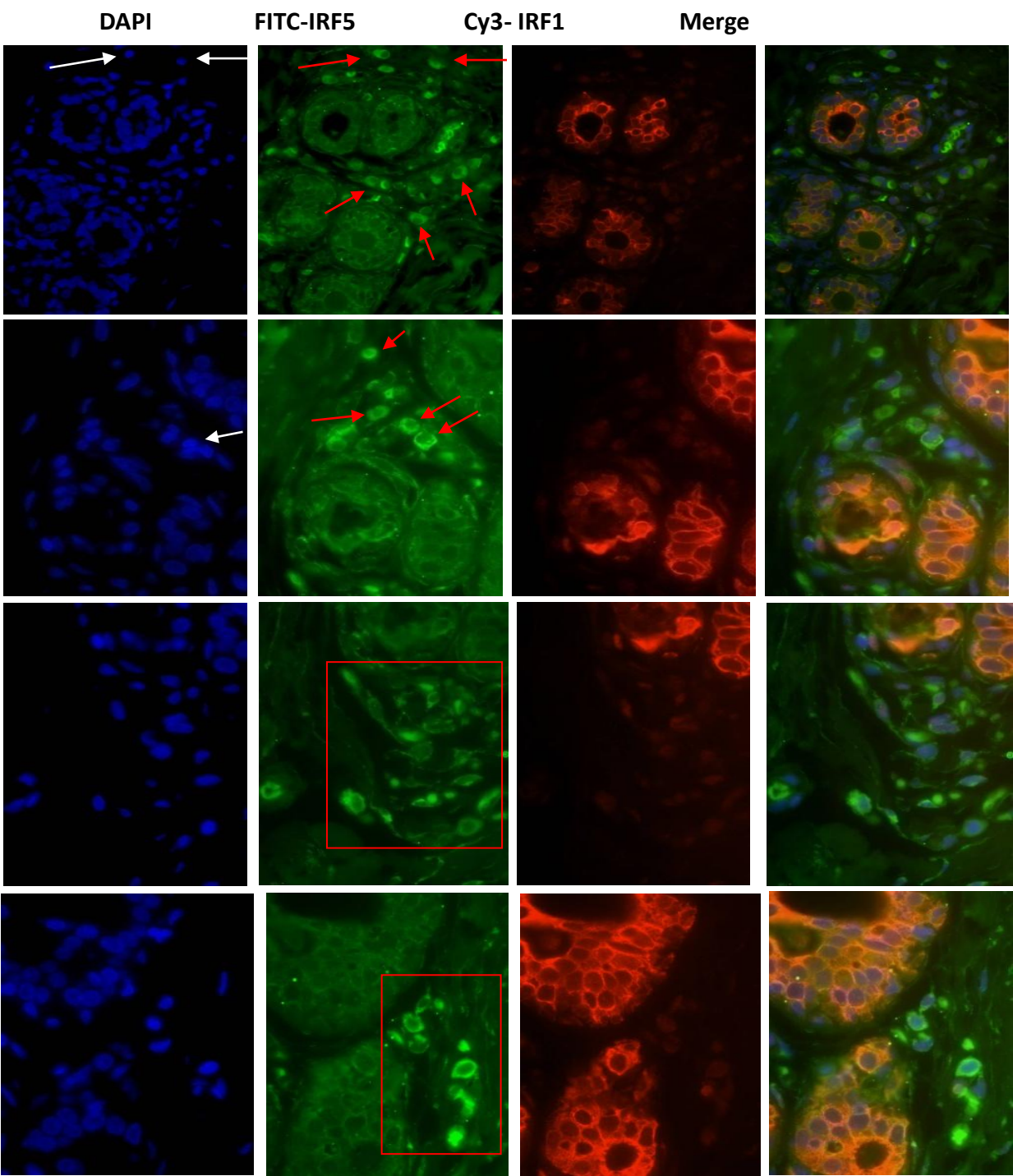

Supplementary Figure 4

Supplement: Additional file 5 — IRF5 is highly expressed in immune/inflammatory cells surrounding normal ducts of IDC patients. IRF1 and IRF5 expression levels were examined by immunofluorescence. [file bcr3053-S5.PDF]
